# Short and long-term outcomes of COVID-19–associated venous thromboembolism: a propensity score–matched cohort study

**DOI:** 10.1007/s11739-025-04042-x

**Published:** 2025-07-03

**Authors:** Rubén Alonso-Beato, Pablo Demelo-Rodríguez, Lucía Ordieres-Ortega, Marina López-Rubio, Marta-Olimpia Lago-Rodríguez, Crhistian-Mario Oblitas, Luis Antonio Alvarez-Sala Walther, Francisco Galeano-Valle

**Affiliations:** 1 Venous Thromboembolism Unit, Department of Internal Medicine. Hospital General, Universitario Gregorio Marañón, Madrid, Spain; 2 Instituto de Investigación Sanitaria Gregorio Marañón, Madrid, Spain; 3 School of Medicine, Universidad Complutense de Madrid, Madrid, Spain; 4 Internal Medicine Department, Hospital Clínico de Santiago, Galicia, Spain; 5 Instituto de Investigación Sanitaria de Santiago, Galicia, Spain

**Keywords:** COVID-19, Anticoagulation, Venous thromboembolism, Pulmonary embolism, Deep vein thrombosis

## Abstract

Venous thromboembolism (VTE) is a recognized complication of SARS-CoV-2 infection, but its clinical features and both sort- and long-term outcomes remain incompletely characterized. We aimed to compare the clinical profile and outcomes of patients with VTE with and without recent COVID-19. We conducted a prospective cohort study including 2012 patients with objectively confirmed VTE. COVID-19–associated VTE was defined as VTE diagnosed within 30 days of a microbiologically confirmed SARS-CoV-2 infection. Clinical characteristics, treatment, and outcomes were compared between groups. Propensity score matching (1:1) and competing risk models were used to adjust for confounding. The primary outcomes-assessed at both 30 days and 365 days-included all-cause mortality, major bleeding, and VTE recurrence. A total of 272 patients (13.5%) had COVID-19–associated VTE. Compared with non-COVID cases, these patients more often had pulmonary embolism, higher D-dimer levels, and greater use of unfractionated heparin. At 30 days, COVID-19 was associated with increased mortality (HR 2.29; 95% CI 1.19–4.40) and major bleeding (HR 2.11; 95% CI 1.06–4.21). At one year, the bleeding risk remained higher (HR 1.54; 95% CI 1.02–2.33), while VTE recurrence was lower (HR 0.34; 95% CI 0.13–0.94). These results were consistent after propensity score matching. COVID-19-associated VTE is linked to worse short-term outcomes, including early mortality and bleeding, and to a persistently elevated bleeding risk at one year. Lower recurrence rates support the consideration of COVID-19 as a transient provoking factor.

**Graphical abstract:**

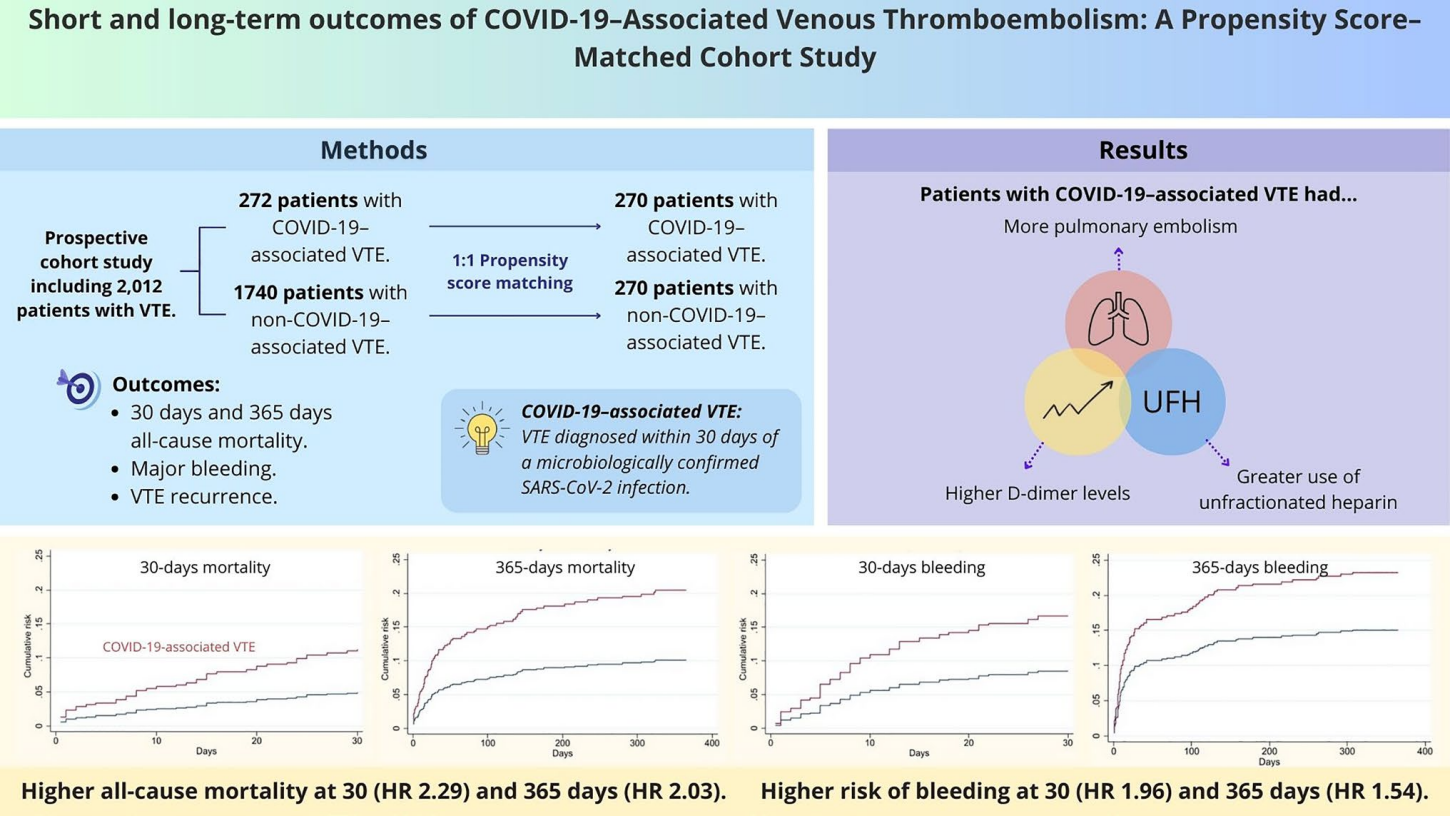

## Introduction

Venous thromboembolism (VTE), encompassing deep vein thrombosis (DVT) and pulmonary embolism (PE), is the third most common cardiovascular disease after coronary artery disease and stroke [1]. In Western countries, its incidence ranges from 0.75 to 2.69 cases per 1000 individuals per year, increasing markedly with age [2]. While the overall incidence is similar between sexes, VTE is more frequent in women of reproductive age and in elderly men [3]. Most events originate in the lower limbs, although other sites -such as the upper limbs or splanchnic and cerebral veins- may also be affected. In recent years, a rise in VTE incidence has been reported, likely related to population aging and an increasing burden of comorbidities [4].

Coronavirus disease 2019 (COVID-19), caused by SARS-CoV-2, has been associated with a heightened risk of VTE, particularly among hospitalized and critically ill patients [5]. The underlying pathophysiological mechanisms are complex and involve endothelial dysfunction, inflammation, platelet activation, and disruption of coagulation and fibrinolysis [6]. Multiple studies conducted during the early months of the pandemic reported a high incidence of VTE in hospitalized patients, especially those in intensive care units, even despite prophylactic anticoagulation [7]. These findings prompted changes in thromboprophylaxis and treatment protocols for patients with COVID-19 and led to the inclusion of COVID-19 as a transient risk factor for VTE in some clinical guidelines [8].

However, the long-term prognosis of patients with COVID-19-associated VTE remains unclear. Some observational studies suggest that these patients have increased short-term mortality, likely driven more by the severity of the viral infection than by the thrombotic event itself [9]. It is also uncertain whether COVID-19-associated VTE entails a different risk of recurrence, bleeding, or post-thrombotic complications, and whether these patients should be managed differently than those with VTE unrelated to COVID-19 [10].

Given these uncertainties, the aim of our study was to compare the short and long-term outcomes of patients diagnosed with VTE with and without recent COVID-19 infection. To minimize confounding factors, we performed a propensity score–matched analysis, enabling us to examine whether the presence of COVID-19 influences the clinical presentation or prognosis of VTE in a real-world setting.

## Methods

### Study design

We performed a prospective, observational, single-center study in a cohort of adult patients (≥ 18 years) with objectively confirmed VTE, diagnosed either in the outpatient or inpatient setting of a tertiary care hospital. Patients were classified according to whether their VTE episode was associated with SARS-CoV-2 infection.

### Study population

We included patients with acute PE or DVT diagnosed either in the emergency department or during hospital admission, who were subsequently followed at the hospital’s VTE unit. VTE was considered COVID-19–associated if it occurred within 30 days of a confirmed SARS-CoV-2 infection, as determined by polymerase chain reaction (PCR) testing.

Patients were consecutively enrolled between March 2020 and March 2024. For the control group (VTE without COVID-19), a matched cohort was selected using 1:1 propensity score matching. Patients with superficial vein thrombosis or incidental VTE (i.e., asymptomatic VTE detected by imaging performed for other reasons) were excluded. In patients with DVT, chest computed tomography was not performed systematically. Instead, imaging to assess for pulmonary embolism was conducted only when clinically indicated, in accordance with standard practice. Similarly, in patients diagnosed with PE, lower-limb ultrasound to assess for DVT was not routinely performed but was ordered based on clinical suspicion.

Patients were clinically followed from the time of VTE diagnosis, both during hospitalization and at subsequent outpatient visits in the dedicated VTE clinic. The minimum follow-up duration was 90 days, or until death if it occurred earlier.

The overall study population consisted of consecutive patients with objectively confirmed VTE. Patients were divided into two groups: those with recent COVID-19 infection (within 30 days of diagnosis) and those without. These groups were subsequently matched using propensity scores to ensure comparability.

### Variables

Clinical, laboratory, and imaging data at the time of VTE diagnosis were collected, along with adverse events occurring during follow-up, including recurrent VTE, bleeding, major bleeding, and all-cause mortality.

### Definitions

COVID-19 severity was assessed using the SEIMC (Sociedad Española de Enfermedades Infecciosas y Microbiología Clínica) score, a validated prognostic tool that estimates 30 day mortality risk in patients hospitalized due to COVID-19 infection. The score incorporates clinical and laboratory variables including age, age-adjusted oxygen saturation, neutrophil-to-lymphocyte ratio, estimated glomerular filtration rate, dyspnea, and sex, and stratifies patients into risk categories with high predictive accuracy [11].

VTE events were classified as provoked or unprovoked according to the definitions of the International Society on Thrombosis and Haemostasis (ISTH). A VTE episode was considered provoked if it occurred in the presence of one or more transient or persistent risk factors, including recent hospitalization or ICU admission, active cancer, recent surgery (within the past two months), or recent immobilization for more than four days. Other provoking factors included recent long-distance travel (over six hours within the preceding three weeks), recent use of hormonal therapy (within the past two months), pregnancy, and recent delivery (within the past two months) [12].

PE severity was assessed using two validated prognostic tools. The first was the simplified Pulmonary Embolism Severity Index (sPESI), which assigns points based on age, cancer, chronic cardiopulmonary disease, heart rate, systolic blood pressure, and oxygen saturation. A score of 0 indicates low risk of 30 day mortality, while a score ≥ 1 or indicates higher risk [13]. In addition, the European Society of Cardiology (ESC) risk stratification model was used. This incorporates clinical parameters including hemodynamic status, cardiac biomarkers (including NT-proBNP and troponins), and right ventricular function on imaging. [14].

Major bleeding was defined according to ISTH definition, as an overt bleeding that is fatal, occurs in a critical area or organ (such as intracranial, intraspinal, intraocular, pericardial, intra-articular, intramuscular with compartment syndrome, or retroperitoneal), causes a fall in hemoglobin level of 2 g/dL or more, or leads to transfusion of two or more units of whole blood or red cells. [15].

VTE recurrence was defined as a new episode of deep vein thrombosis or pulmonary embolism, confirmed by objective imaging and occurring after the initial event, during follow-up.

### Statistical analysis

Descriptive statistics were performed for the overall cohort and stratified by COVID-19 status. Categorical variables were presented as frequencies and percentages, while continuous variables were expressed as mean ± standard deviation or median [P25–P75], depending on distribution assessed using the Shapiro–Wilk and Shapiro-Francia tests. Group comparisons were performed using the chi-squared test or Fisher’s exact test for categorical variables, and Student’s t-test or the Mann–Whitney U test for continuous variables, as appropriate.

Cox proportional hazards models were used to assess the association between COVID-19 infection and the occurrence of complications at 30 days and 12 months (all-cause mortality, major bleeding, and recurrent VTE), reporting hazard ratios (HRs) with 95% confidence intervals (CIs). To account for the competing risk of death, sub-hazard ratios (sHRs) were also estimated using Fine and Gray’s competing risk regression models. Event incidence rates were reported as events per 100 person-years, and incidence rate ratios (IRRs) were calculated to compare outcomes between groups.

To compare outcomes between patients with and without recent SARS-CoV-2 infection, we used COVID-19 status as the independent variable of interest. Propensity score matching was applied to minimize confounding related to VTE severity and baseline clinical complexity. A 1:1 propensity score matching was conducted using a greedy nearest-neighbor algorithm without replacement and a caliper width of 0.05. Variables included in the model were selected based on clinical and statistical relevance. A standardized mean difference < 10% was considered indicative of adequate covariate balance.

The variables used to construct the propensity score model included: age, sex, cancer, hospitalization, provoked vs. unprovoked VTE (excluding COVID-19), recent major bleeding, DVT vs. PE presentation, ESC risk classification (in PE), thrombophilia, kidney failure (eGFR < 60 mL/min), and thrombocytopenia (< 100,000/mm^3^).

All regression analyses were repeated in the matched cohort. A two-sided p-value < 0.05 was considered statistically significant. All analyses were performed using IBM SPSS Statistics v25.0^®^ (IBM Corp. ©, Armonk, NY) and R software v4.0.5^®^ (R Core Team ©, 2021).

### Ethical considerations

This study was conducted in accordance with the Declaration of Helsinki and Good Clinical Practice guidelines. This prospective study is part of a local prospective registry approved by the Ethics Committee in 2015. A subsequent approval (reference 23/2022) was obtained in 2022 due to a change in the principal investigator, with no changes to the study protocol. As an observational study involving no modifications to standard clinical care, it was deemed to pose no additional risk to participants. All procedures performed in this study involving human participants were in accordance with the ethical standards of the institutional and/or national research committee and with the 1964 Helsinki declaration and its later amendments. This study did not involve any experiments on animals. Informed consent was obtained from all individual participants included in the study.

## Results

A total of 2,012 patients with confirmed VTE were included, of whom 272 (13.5%) had a recent SARS-CoV-2 infection. Patients with COVID-19–associated VTE were more frequently male (64.3% vs. 50.7%, p < 0.001), had a lower prevalence of smoking and chronic kidney disease, and were more often diagnosed during hospitalization. Most VTE in the COVID-19 group were provoked (87.9% vs. 56.8%, p < 0.001), excluding SARS-CoV-2 infection itself as a provoking factor in this analysis. No significant differences were observed in median age, obesity, or most cardiovascular comorbidities (Table 1).

**Table 1:** Demographics, comorbidities, risk factors, clinical presentation, laboratory data, and treatment characteristics in patients with and without COVID-19–associated VTE

Variable	COVID-19 associated VTE (n = 272)	Non-COVID-19 VTE (n = 1740)	p-value
Demographics and comorbidities			
Male sex (%)	64.3	50.7	** < 0.001**
Age, median (IQR)	66.5 (55–76)	68 (53–79)	0.590
Obesity (BMI > 30) (%)	36.4	34.1	0.446
Hypertension (%)	46.3	48.1	0.597
Diabetes (%)	14.7	14.6	0.965
Dyslipidemia (%)	30.9	30.1	0.783
Smoking (%)	4.4	16.1	** < 0.001**
Ischemic heart disease (%)	9.2	5.0	**0.005**
Stroke (%)	8.1	7.1	0.570
Peripheral artery disease (%)	1.5	2.5	0.288
Heart failure (%)	4.4	6.8	0.131
Atrial fibrillation (%)	3.3	2.8	0.652
Chronic kidney disease (%)	5.9	12.4	**0.002**
Recent major bleeding (%)	2.9	4.6	0.214
Risk factors			
Unprovoked VTE (excluding COVID-19) (%)	12.1	43.2	** < 0.001**
Diagnosis during hospitalization (%)	52.6	12.8	** < 0.001**
ICU stay (%)	18.4	1.2	** < 0.001**
Active cancer (%)	16.5	20.4	0.138
Recent surgery (%)	6.3	11.3	**0.012**
Immobilization (%)	77.2	28.3	** < 0.001**
Recent travel (%)	0.4	2.3	**0.037**
Hormone therapy (%)	3.0	5.7	0.061
Pregnancy (%)	0.0	0.5	0.608
Recent delivery (%)	0.4	0.5	1.000
Previous VTE (%)	5.2	7.9	0.112
Family history of VTE (%)	1.5	7.6	** < 0.001**
Thrombophilia (%)	3.3	5.4	0.145
Diagnosis			
DVT (with/without PE) (%)	36.0	56.2	** < 0.001**
PE (with/without DVT) (%)	77.6	56.1	** < 0.001**
Both DVT and PE (%)	18.0	18.7	0.776
In patients with PE			
Tachycardia > 100 bpm (%)	31.8	27.8	0.245
Systolic blood pressure < 90 mmHg (%)	6.6	4.3	0.147
Respiratory failure (SpO2 < 90%) (%)	28.1	30.8	0.613
sPESI ≥ 1 point (%)	61.6	67.1	0.126
ESC risk classification			
Low (%)	23.2	20.8	0.435
Intermediate-low (%)	60.2	58.4	0.632
Intermediate-high (%)	10.0	16.5	**0.017**
High (%)	7.6	5.3	0.201
RV hypokinesia on echocardiography (%)	33.3	36.1	0.505
Central PE location (%)	17.1	31.6	** < 0.001**
Subsegmental location only (%)	17.5	7.1	** < 0.001**
Other locations (%)	65.4	61.4	0.274
In patients with DVT			
Lower limbs (%)	88.7	87.3	0.699
Proximal DVT (%)	54.7	79.0	** < 0.001**
Upper limbs (%)	6.3	7.5	0.838
Laboratory			
Anemia (%)	31.6	27.5	0.163
Thrombocytopenia < 100,000/mm3 (%)	4.0	2.5	0.154
Kidney failure (eGFR < 60 mL/min) (%)	15.9	24.1	**0.003**
Elevated D-dimer > 300 ng/mL (%)	94.1	78.2	** < 0.001**
D-dimer, ng/mL, median (IQR)	2987 (1610–7834)	2472.5 (1115–5840)	** < 0.001**
In patients with PE			
Elevated troponin (%)	30.1	38.5	**0.048**
Elevated NT-proBNP > 600 pg/mL (%)	39.2	44.2	0.264
NT-proBNP, pg/mL, median (IQR)	339.5 (118–1235)	430 (140–1703)	0.200
Treatment			
Acute phase (first 10 days)			
Fibrinolysis (%)	3.7	1.7	**0.032**
LMWH (%)	86.0	87.7	0.439
UFH (%)	19.5	7.9	** < 0.001**
DOAC (%)	8.5	7.4	0.536
Long-term (day 10 to 90)			
LMWH (%)	20.2	21.3	0.679
DOAC (%)	60.3	71.0	** < 0.001**
VKA (%)	8.5	10.9	0.229
IVC filter (%)	3.3	3.1	0.856

*IQR* interquartile range, *BMI* body mass index, *ICU* intensive care unit, *VTE* venous thromboembolism, *DVT* deep vein thrombosis, *PE* pulmonary embolism, *sPESI* simplified Pulmonary Embolism Severity Index, *ESC* European Society of Cardiology, *RV* right ventricle, *eGFR* estimated glomerular filtration rate, *NT-proBNP* N-terminal pro–B-type natriuretic peptide, *LMWH* low molecular weight heparin, *UFH* unfractionated heparin, *DOAC* direct oral anticoagulant, *VKA* vitamin K antagonist, *IVC* inferior vena cava

Bold values indicate statistically significant results (p<0.05)

In terms of clinical presentation, PE was more common in the COVID-19 group (77.6% vs. 56.1%, p < 0.001), while isolated DVT was less frequent (36.0% vs. 56.2%, p < 0.001). Among patients with PE, those with COVID-19 more often had subsegmental emboli (17.5% vs. 7.1%, p < 0.001) and less frequently central emboli (17.1% vs. 31.6%, p < 0.001). No significant differences were observed in vital signs or rates of hypoxemia.

COVID-19 patients also had higher D-dimer levels at diagnosis (median 2,987 ng/mL vs. 2,472 ng/mL, p < 0.001), a lower prevalence of kidney failure (15.9% vs. 24.1%, p = 0.003), and similar rates of anemia and thrombocytopenia (Table 1).

Regarding anticoagulant therapy, patients with COVID-19 more frequently received unfractionated heparin during the acute phase (19.5% vs. 7.9%, p < 0.001) and were less likely to be treated with direct oral anticoagulants during follow-up (60.3% vs. 71.0%, p < 0.001) (Table 1).

Among patients with COVID-19–associated VTE, disease severity was assessed using the SEIMC score. Most patients were classified as high or very high SEIMC risk, with 27.2% and 41.2% falling into these categories, respectively. Only a minority were categorized as low (5.5%) or moderate risk (26.1%).

Outcome analysis showed significantly higher all-cause mortality in the COVID-19 group at both 30 days (IRR 3.09; HR 3.10; p < 0.001) and 365 days (IRR 1.82; HR 2.39; p < 0.001). Bleeding complications were also more frequent among COVID-19 patients, particularly within the first 30 days, including any bleeding (IRR 2.30; HR 2.30; p < 0.001) and major bleeding (IRR 2.72; HR 2.67; p < 0.001) (Table 2).

**Table 2:** Incidence rates, incidence rate ratios, and risk estimates for all-cause mortality, bleeding, major bleeding, and recurrent VTE in patients with and without COVID-19 associated VTE

Outcome	Follow-up	COVID rate	Non-COVID rate	Overall rate	Rate ratio (95% CI)	p	Cox HR (95% CI)	p	Fine & Gray SHR (95% CI)	p
Mortality	30 days	129.33	41.83	53.29	3.091 (1.882–4.960)	** < 0.001**	3.100 (1.994–4.818)	** < 0.001**	–	–
	365 days	21.31	12.80	14.47	1.820 (1.287–2.535)	**0.006**	2.392 (1.739–3.291)	** < 0.001**	–	–
Bleeding	30 days	201.18	87.28	102.19	2.304 (1.582–3.299)	** < 0.001**	2.300 (1.623–3.275)	** < 0.001**	2.305 (1.627–3.267)	** < 0.001**
	365 days	27.11	19.48	20.69	1.392 (1.021–1.871)	**0.030**	1.795 (1.341–2.404)	** < 0.001**	1.795 (1.339–2.408)	** < 0.001**
Major bleeding	30 days	119.74	44.00	53.91	2.721 (1.636–4.400)	**0.001**	2.674 (1.679–4.260)	** < 0.001**	2.674 (1.683–4.248)	** < 0.001**
	365 days	13.32	9.46	10.08	1.406 (0.892–2.151)	0.117	–	–	–	–
Recurrence	30 days	4.78	10.81	10.03	0.442 (0.010–2.877)	0.467	0.424 (0.056–3.215)	0.407	0.424 (0.056–3.204)	0.406
	365 days	1.90	6.58	5.83	0.289 (0.076–0.772)	**0.004**	0.344 (0.125–0.941)	**0.038**	0.344 (0.126–0.938)	**0.037**

Rates are expressed per 100 person-years

*CI* confidence interval, *HR* hazard ratio (Cox model), *SHR* sub-hazard ratio (Fine & Gray model for competing risks)

Bold values indicate statistically significant results (p<0.05)

At 12 months, there were no statistically significant differences in the incidence of major bleeding between COVID-19 and non–COVID-19 patients (HR 1.45; 95% CI 0.81–2.60). In contrast, VTE recurrence at 12 months was significantly lower in the COVID-19 group (IRR 0.29; HR 0.34; p = 0.038) (Table 2).

**Fig. 1:**
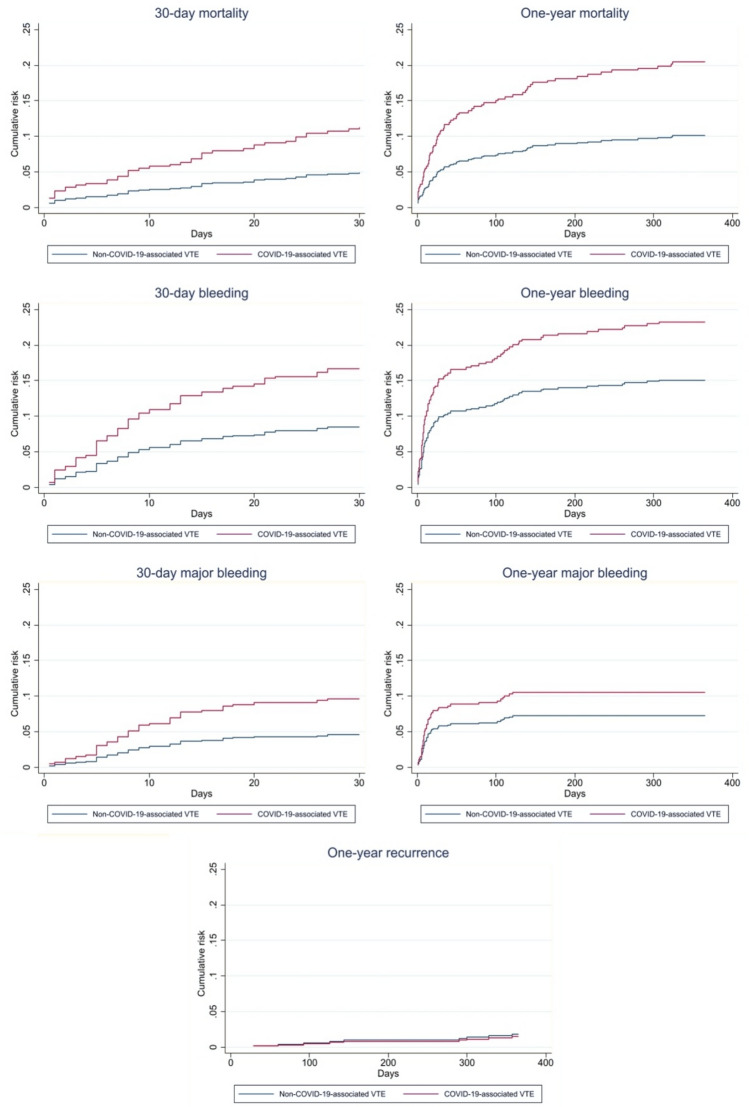
Cumulative risk of clinical outcomes at 30 days and one year after propensity-score matching

After propensity score matching (270 patients per group), baseline characteristics were well balanced, with standardized mean differences below 10% for all variables (Tables 3 and 4). In the matched cohort, COVID-19 remained significantly associated with higher all-cause mortality at both 30 and 365 days (HRs 2.29 and 2.03, respectively). The risk of bleeding also remained elevated in the COVID-19 group, both at 30 (HR 1.96) and 365 days (HR 1.54). In contrast, there were no significant differences in VTE recurrence rates between the two groups after matching (HR 0.80; p = 0.735) (Table 5 and Fig. 1).

**Table 3:** Baseline characteristics before and after propensity score matching

Variable	Before Matching COVID-19 associated VTE (n = 272)	Before Matching Non-COVID-19 VTE (n = 1740)	SMD Before (%)	After Matching COVID-19 associated VTE (n = 270)	After Matching Non-COVID-19 VTE (n = 270)	SMD After (%)
Male sex	175 (64.34%)	882 (50.69%)	27.8	173 (64.07%)	165 (61.11%)	6.1
Age (years, median [IQR])	66.5 (55–76)	68 (53–79)	− 1.4	66.5 (55–76)	66 (54–78)	− 1.9
Recent major bleeding (last month)	8 (2.94%)	80 (4.60%)	− 8.6	8 (2.96%)	12 (4.44%)	− 7.8
Provoked event (excluding COVID-19)	239 (87.87%)	989 (56.84%)	73.9	237 (87.78%)	231 (85.56%)	6.5
Hospitalization	143 (52.57%)	222 (12.77%)	93.6	142 (52.59%)	150 (12.77%)	− 5.9
Cancer	45 (16.54%)	355 (20.40%)	− 9.9	44 (16.30%)	52 (55.56%)	− 7.7
Thrombophilia	9 (3.31%)	94 (5.40%)	− 10.2	9 (3.33%)	9 (3.33%)	0.0
DVT	98 (36.03%)	977 (56.15%)	− 41.1	98 (36.30%)	108 (40.00%)	− 7.6
PE	211 (77.57%)	976 (56.09%)	46.8	209 (77.41%)	210 (77.78%)	− 0.8
ESC risk in PE			− 16.3			− 9.5
• Low	49 (23.22%)	203 (20.80%)		49 (23.44%)	47 (22.38%)	
• Intermediate-low	127 (60.19%)	570 (58.40%)		126 (60.29%)	132 (62.86%)	
• Intermediate-high	21 (9.95%)	161 (16.50%)		20 (9.57%)	22 (10.48%)	
• High	16 (7.58%)	52 (5.33%)		16 (7.66%)	12 (5.71%)	
Kidney failure (eGFR < 60 mL/min)	43 (15.87%)	418 (24.08%)	− 20.6	43 (15.93%)	38 (14.07%)	5.1
Thrombocytopenia (< 100,000/mm^3^)	11 (4.04%)	44 (2.53%)	8.4	10 (3.70%)	8 (2.96%)	4.1

*ESC* European Society of Cardiology, *eGFR* estimated glomerular filtration rate, *SMD* standardized mean difference, *IQR* interquartile range

**Table 4:** Baseline characteristics and balance diagnostics before and after propensity score matching

Variable	Standardized mean difference (%) Before matching	After matching	Bias reduction (%)
Male sex	27.8	6.1	77.9
Age (years, median [IQR])	− 1.4	− 1.9	12.3
Recent major bleeding (last month)	− 8.6	− 7.8	10.7
Provoked event (excluding COVID-19)	73.9	6.5	92.8
Hospitalization	93.6	− 5.9	92.6
Cancer	− 9.9	− 7.7	28.9
Thrombophilia	− 10.2	0.0	100
Deep vein thrombosis	− 41.1	− 7.6	81.6
Pulmonary embolism	46.8	− 0.8	98.3
ESC risk in PE	− 16.3	− 9.5	41.7
Kidney failure (eGFR < 60 mL/min)	− 20.6	5.1	77.5
Thrombocytopenia (< 100,000/mm^3^)	8.4	4.1	51.4
Global matching diagnostics			
Pseudo R-squared	0.216	0.011	–
LR chi-square	342.89	7.93	–
Chi-square p-value	< 0.001	0.790	–
Mean bias (%)	30.0	5.2	–
Median bias (%)	18.5	5.7	–
B (bias)	129.7	24.3	–
R (variance ratio)	1.42	0.96	–
% of covariates with variance out of range	50%	0%	–

*IQR* interquartile range, *ESC* European society of cardiology, *PE* pulmonary embolism, *eGFR* estimated glomerular filtration rate, *LR* likelihood ratio

**Table 5:** Risk estimates for mortality, bleeding, major bleeding, and recurrence after propensity-score matching

Outcome	Follow-up	Cox HR (95% CI)	p	Fine & Gray SHR (95% CI)	p
Mortality	30 days	2.285 (1.188–4.397)	**0.013**		
	365 days	2.026 (1.261–3.255)	**0.003**		
Bleeding	30 days	1.961 (1.170–3.285)	**0.010**	1.961 (1.170–3.285)	**0.011**
	365 days	1.541 (1.021–2.327)	**0.039**	1.541 (1.022–2.324)	**0.039**
Major bleeding	30 days	2.113 (1.061–4.206)	**0.033**	2.113 (1.060–4.209)	**0.033**
	365 days	1.450 (0.808–2.604)	0.213	1.450 (0.808–2.604)	0.213
Recurrence	30 days	–	–	–	–
	365 days	0.796 (0.213–2.966)	0.735	0.796 (0.214–2.962)	0.734

*CI* confidence interval, *HR* hazard ratio (Cox model), *SHR* sub-hazard ratio (Fine & Gray model for competing risks)

Bold values indicate statistically significant results (p<0.05)

## Discussion

In this single-center cohort study of over 2,000 patients with objectively confirmed VTE, we found that VTE associated with recent SARS-CoV-2 infection was linked to a distinct clinical profile, increased early mortality, and a higher risk of bleeding, but a lower rate of recurrence compared to VTE not related to COVID-19. These associations remained consistent after adjustment using propensity score matching and competing risk models. These findings align with and expand upon prior studies examining thrombotic complications in the context of COVID-19 [16].

Similar to previous reports, patients with COVID-19–associated VTE were more likely to be male and to have recently been hospitalized, and they presented more frequently with PE and elevated D-dimer levels [17]. This phenotype has been consistently described in observational studies and registries [18, 19], supporting the notion that COVID-19 may induce a prothrombotic state via mechanisms beyond traditional risk factors, including endothelial injury, hyperinflammation, and in situ pulmonary thrombosis [20, 21]. Beyond the specific context of COVID-19, it is well recognized that prolonged inflammation resulting from severe infection or sepsis can profoundly disturb the balance between coagulation and fibrinolysis. This occurs through multiple interrelated mechanisms, including decreased levels of natural anticoagulants such as antithrombin and protein C, and increased concentrations of plasminogen activator inhibitor-1 (PAI-1), a potent antifibrinolytic protein. These changes collectively promote sustained activation of the coagulation cascade while inhibiting fibrinolysis, ultimately increasing the risk of thrombotic events [6].

Our study reinforces findings from the RIETE registry, where COVID-19–associated PE was more often provoked and was associated with increased early mortality [22]. The significantly higher all-cause mortality observed in patients with COVID-19–associated VTE likely reflects the acute severity of SARS-CoV-2 infection and VTE as a complication. Prior studies have shown that the occurrence of VTE in the context of COVID-19 markedly increases the risk of death both in outpatients (HR 4.42; 95% CI 3.07–6.36) and hospitalized patients (HR 1.63; 95% CI 1.39–1.90), thus underscoring the prognostic implications of thrombotic events in this setting [9]. These findings highlight the importance of early identification and management of VTE in COVID-19 patients, particularly during the acute phase of the infection when the risk of adverse outcomes is highest.

Bleeding risk was significantly higher among patients with COVID-19–associated VTE, particularly during the early follow-up. This finding is consistent with previous studies estimating a 3.9% incidence of major bleeding among hospitalized patients with COVID-19–related VTE (95% CI 1.2–7.9) [22]. Importantly, the occurrence of major bleeding events has been shown to more than double 30-day mortality in these patients and constitutes an independent predictor of death [23]. These findings highlight the delicate balance between thrombosis and bleeding in COVID-19 patients receiving anticoagulation and underscore the importance of tailored risk assessment in patients with COVID-19–associated VTE, particularly in terms of bleeding risk and optimal duration of anticoagulation.

Regarding recurrence risk, our findings are consistent with previous evidence suggesting that COVID-19–associated VTE behaves as a transiently provoked event [24]. In our cohort, the 12-month recurrence rate was lower than in non–COVID-19 patients. This aligns with early prospective studies, such as one including 48 hospitalized patients with COVID-19–related VTE, which reported no recurrences after one year of follow-up [25]. Subsequent studies have estimated recurrence rates ranging between 2.2 and 4.2 events per 100 person-years in patients whose anticoagulation was stopped after the acute phase [26]. These figures are notably lower than recurrence rates reported in non–COVID-19 VTE, where a recent meta-analysis found rates of 5.8 per 100 person-years for VTE provoked by a non-surgical risk factor and 10.3 per 100 person-years for unprovoked events [27]. These differences further support the concept that COVID-19–associated VTE has a lower long-term thrombotic risk and justify current recommendations favoring short-course anticoagulation (typically 3–6 months) in the absence of persistent risk factors.

These findings should also be interpreted in the context of the evolving nature of the COVID-19 pandemic. A recent study using data from the RIETE registry compared outcomes of COVID-19–associated VTE before and after the widespread introduction of vaccination, and reported a significant reduction in 90-day mortality (adjusted HR 2.27; 95% CI 1.18–4.38) and major bleeding (adjusted HR 2.91; 95% CI 1.08–7.84) in the post-vaccine period (2021–2022), compared to the early phase of the pandemic in 2020. These results suggest that vaccination and improved management strategies may have contributed to better outcomes in more recent cohorts [28].

The strengths of this study include a large, well-characterized cohort with objective VTE diagnosis, comprehensive outcome assessment, and robust adjustment for confounding using propensity score matching. Nevertheless, several limitations must be considered. First, as a single-center study, external generalizability may be limited. Second, data on viral variants and vaccination status were not consistently available, which could impact interpretation. Third, although matched, residual confounding is inherent to observational designs and remains as a limitation. Fourth, we were unable to fully adjust for the degree of respiratory failure due to incomplete data on oxygen supplementation or ventilatory support, which may have influenced both the risk of complications and clinical decision-making. Finally, while our study focuses on COVID-19–associated VTE, we recognize that many of the mechanisms implicated—such as inflammation-driven endothelial damage and procoagulant changes—are shared by other acute infections. The higher risk of adverse outcomes observed in our COVID-19 cohort may therefore, at least in part, reflect common pathways of thromboinflammation that are not exclusive to SARS-CoV-2 infection. Future comparative studies in patients with non-COVID infections and VTE could help delineate shared and specific features of thrombosis in systemic infectious states.

Overall, our findings contribute to the growing evidence that COVID-19–associated VTE is a clinically distinct entity, with implications for personalized management strategies, particularly in the early post-diagnosis period. While early mortality and bleeding risk are clearly elevated, recurrence appears less concerning in the long term.

## Conclusion

In conclusion, COVID-19–associated VTE is characterized by a distinct clinical profile, higher early mortality, increased bleeding risk, and lower recurrence rates compared to non–COVID-19 VTE. These findings support the notion that COVID-19–associated thrombosis represents a unique clinical scenario, particularly during the initial phase following diagnosis.
